# Focal Myopericarditis as a Rare but Important Differential Diagnosis of Myocardial Infarction; a Case Series

**Published:** 2016

**Authors:** Younes Nozari, Masih Tajdini, Mehdi Mehrani, Rosa Ghaderpanah

**Affiliations:** Tehran Heart Center, Tehran University of Medical Sciences, Tehran, Iran.

**Keywords:** Coronary angiography, electrocardiography, myocardial infarction, myocarditis, emergency medicine

## Abstract

Distinguishing ST-elevation myocardial infarction (STEMI) differential diagnoses is more challenging. Myopericarditis is one of these differentials that results from viral involvement of myocardium and pericardium of the heart. Myopericarditis in focal form can mimic acute STEMI in its electrocardiogram (ECG) features and elevated cardiac enzymes.

Myocarditis patients may face thrombolytic related complications such as intracranial bleeding, myocardial rupture, and hemorrhagic cardiac tamponade. Furthermore, re-administration of streptokinase (a common thrombolytic agent in our country) is banned for at least six months of previous administration; however, it can save patients’ lives in emergency conditions such as massive pulmonary embolism. It seems that, when dealing with a young patient presenting to emergency department with acute chest pain and ST segment elevation on ECG, we should consider focal myocarditis as an important but rare differential diagnosis of STEMI. In this report, we describe three cases of focal myocarditis, primarily misdiagnosed as STEMI.

## Introduction

Determining the necessity and timing of urgent revascularization are important matters in patients who present with chest pain accompanied by ST segment elevation and possible myocardial infarction. Distinguishing differential diagnoses of ST-elevation myocardial infarction (STEMI) is even more challenging.

Myopericarditis is one of these differentials that results from viral involvement of myocardium and pericardium of the heart. Pleuritic chest pain, fatigue and decreased exercise capacity with history of a febrile syndrome are its common manifestations (1). Myopericarditis in focal form can mimic acute STEMI in its electrocardiogram (ECG) features and elevated cardiac enzymes (2). 

Endo-myocardial biopsy (EMB) with the histological Dallas criteria is the gold standard for diagnosing myocarditis. However, when we have focal involvement, EMB is limited by high variability and sampling error (3, 4). Recently, cardiac magnetic resonance (CMR) with gadolinium has been introduced as an accurate technique to differentiate acute myocarditis from acute myocardial infarction (5, 6). Myocarditis patients may face thrombolytic related complications such as intracranial bleeding, myocardial rupture, and hemorrhagic cardiac tamponade. Furthermore, re-administration of streptokinase (a common thrombolytic agent in our country) is banned for at least six months of previous administration; however, it can save patients’ lives in emergency conditions such as massive pulmonary embolism, ischemic stroke and STEMI (7, 8). In this report, we describe three cases of focal myocarditis, primarily misdiagnosed as STEMI.

## Case presentation

All three cases reported in this series are young patients presented to emergency department following 2-3 hours of constant severe retrosternal chest pain and ECG findings implying STEMI. Table 1 summarizes clinical and diagnostic findings of patients. Figure 1 shows 12 leads ECGs of cases 1 to 3, respectively. All three patients were sent to catheterization laboratory and were diagnosed with focal myocarditis, based on collective evidence. Patients were treated with 600 mg Ibuprofen three times a day along with 0.5 mg Colchicine twice daily for two weeks. Abnormal ECG findings were reversed after three days of treatment. Ejection fractions of patients at one-month follow up echocardiography were 50-55%, 50%, and 50% for cases 1 – 3, respectively. No evidence of regional wall motion abnormality was detected on one-month follow up echocardiography of the studied patients. For all of these patients, secondary causes of myopericarditis were assessed. All serologic markers were in normal range.

**Table 1:** Clinical and diagnostic findings of studied patients

**Findings**	**Case 1**	**Case 2**	**Case 3**
**Age (years) **	41	20	26
**Sex**	Male	Male	Female
**Cardiac risk factor**	Cigarette smoker	Negative	Cigarette smoker
**History of febrile disease**	URI 2 weeks ago	URI 4 days ago	Negative
**Physical examination**	Normal	Normal	Normal
**Vital signs**			
Systolic BP (mmHg)	110	120	110
Diastolic BP (mmHg)	70	80	75
Pulse rate / minute	90	75	75
Respiratory rate / minute	24	22	20
Temperature (C)	37	37.3	37.4
**Electrocardiographic**			
ST elevation	ΙΙ, ΙΙΙ, aVF	Ι, aVL	ΙΙ, ΙΙΙ, aVF
ST depression	I, aVL	ΙΙΙ, aVF	aVL
**Echocardiographic**			
Wall motion abnormality	Base and mid-inferior	Posterior	No
Ejection fraction (%)	45	35-40	50
**hs-cTnT (pg/ml)** ^#^			
Baseline	540	83	177
3 hours later	711	1026	1395
**Angiography**	Normal	Normal	Normal

URI:Upper respiratory tract infection.

* Normal range: ˂ 14 pg/ml.

# hs-cTnT: high-sensitive cardiac troponin T.

**Figure 1:**
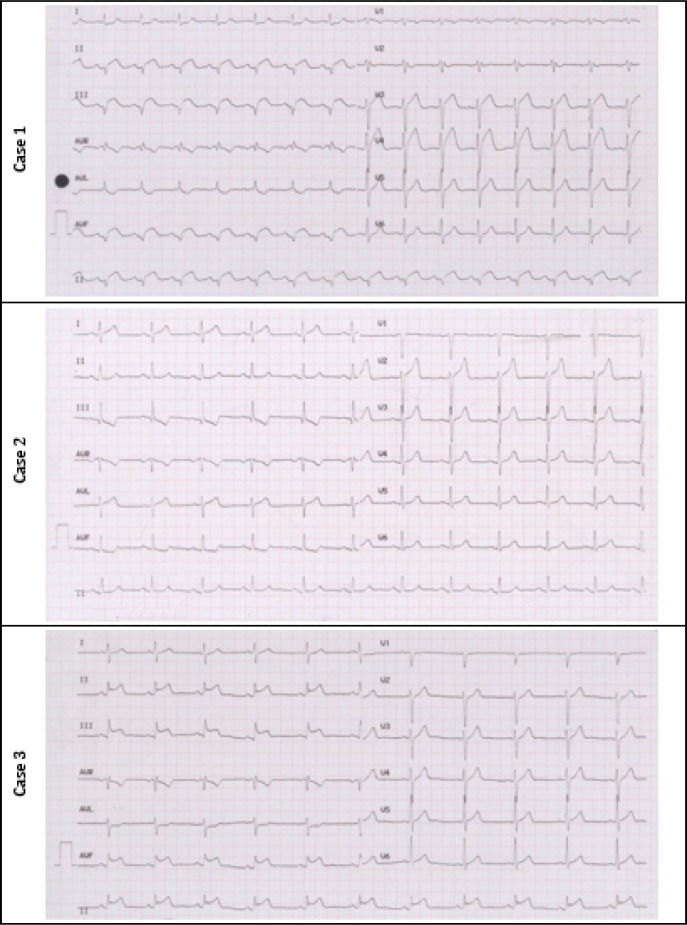
12-leads electrocardiogram of reported patients

## Discussion

Chest pain accompanied by elevated cardiac enzymes and normal coronary arteries are in favor of myocarditis diagnosis. Although, our patients were young and had history of preceding viral illness, ST- segment elevation, which supports involvement of one vessel territory, raised the possibility of an acute coronary syndrome. 

Myocarditis is caused by diffuse inflammatory process of immune system in response to an etiologic factor. The etiologic factor is usually one of the following: infectious, post viral autoimmune-related, autoimmune-mediated (lupus myocarditis, giant-cell myocarditis [GCM]) or drug-associated (hypersensitivity myocarditis, toxic myocarditis). Overlap of virus-mediated damage, inflammation and autoimmune dysregulation leads to myocardial injury and necrosis. Thus, in generalized form of myocarditis, left ventricular dysfunction occurs and may proceed to end stage heart failure (9). It seems that in focal myocarditis just a small section of myocardium is involved, so there is less myocardial damage and it is reversible.

**Differential diagnosis**

STEMI is the most important differential diagnosis of focal myocarditis. Eosinophilic endomyocardial disease (Loeffler syndrome) is another differential diagnosis that is characterized by extensive necrosis, mural and intravascular thrombi without pathologic or clinical myocarditis. Hypersensitivity myocarditis is an important differential diagnosis that should be ruled out, because cardiac function may improve by cessation of offending drug. cardiac sarcoidosis (CS), which is characterized by epithelioid granulomas formation, should also be considered in differential diagnosis (10).

**Emergency department management**

Cardiac monitoring for detecting dysrhythmias is necessary for these patients. Supplemental oxygen and fluid should be considered based on the patient's condition. Myocarditis with reduced ejection fraction should be approached the same as other causes of congestive heart failure (CHF). Sympathomimetic drugs should be avoided due to their role in increasing the extent of myocardial necrosis and mortality. In addition, Beta-blockers should not be administered in the acutely decompensated phase of illness. Temporary pacemaker placement is required in symptomatic high degree atrioventricular blocks but very few patients may need permanent pacer or implantable cardioverter defibrillator (ICD) placement (11).

**Diagnostic approach**

Definitive diagnosis can be confirmed by different techniques. Endo-myocardial biopsy is the gold standard method with high specificity and low sensitivity. However, complications such as myocardial perforation and death have been reported. In focal myocarditis, myocardial biopsy is not so helpful because the involvement is local and biopsy may be taken from the unaffected part (7, 8). Edema and myocyte inflammation can be detected by cardiac magnetic resonance imaging (MRI). Unlike MI, in which sub-endocardial enhancement is the characteristic finding, in myocarditis enhancement is originated from epicardium with sub-endocardial sparing. In a study, biopsy specimens taken from areas of gadolinium enhancement had myocarditis features on pathologic findings in 90% of cases (4, 5). In addition, Cardiac computed tomography (CT) scan can be used for diagnosis. Recent studies showed that findings were similar between cardiac CT and MRI. CT has some superiority in comparison to MRI, including feasibility of coronary artery anatomy investigation, better evaluation of myocardial inflammation and lower costs (11, 12). Speckle tracking echocardiogram (STE) is a precise method for evaluation of myocarditis patients. In a study, Strain (S) and Strain Rate (SR) imaging were compared in acute myocarditis patients with preserved left ventricular ejection fraction. Septal thickness, left ventricular end-systolic dimension (LVESD), and ejection fraction were different between them. Therefore, STE technique helps us thoroughly evaluate patients who initially present with acute myocarditis (13). In addition to cardiac injury markers, the level of brain natriuretic peptide (BNP) can also be measured. 

**Prognosis**

In contrast to generalized myocarditis that can proceed to congestive heart failure and death due to arrhythmia or severe left ventricular dysfunction, it seems that prognosis is better in focal form (-). High concentrations of BNP are correlated with poor prognosis in patients with myocarditis (14).

## Conclusion

When dealing with a young patient presenting to emergency department with acute chest pain and ST segment elevation on ECG, we should consider focal myocarditis as an important but rare differential diagnosis of STEMI.
